# The Role of Staphylococcal Biofilm on the Surface of Implants in Orthopedic Infection

**DOI:** 10.3390/microorganisms10101909

**Published:** 2022-09-26

**Authors:** Yu Lu, Wei-jie Cai, Zun Ren, Pei Han

**Affiliations:** Shanghai Sixth People’s Hospital Affiliated to Shanghai Jiao Tong University School of Medicine, Shanghai 200025, China

**Keywords:** biofilm, bacterial adhesion, implant-associated infection

## Abstract

Despite advanced implant sterilization and aseptic surgical techniques, implant-associated infection remains a major challenge for orthopedic surgeries. The subject of bacterial biofilms is receiving increasing attention, probably as a result of the wide acknowledgement of the ubiquity of biofilms in the clinical environment, as well as the extreme difficulty in eradicating them. Biofilm can be defined as a structured microbial community of cells that are attached to a substratum and embedded in a matrix of extracellular polymeric substances (EPS) that they have produced. Biofilm development has been proposed as occurring in a multi-step process: (i) attachment and adherence, (ii) accumulation/maturation due to cellular aggregation and EPS production, and (iii) biofilm detachment (also called dispersal) of bacterial cells. In all these stages, characteristic proteinaceous and non-proteinaceous compounds are expressed, and their expression is strictly controlled. Bacterial biofilm formation around implants shelters the bacteria and encourages the persistence of infection, which could lead to implant failure and osteomyelitis. These complications need to be treated by major revision surgeries and extended antibiotic therapies, which could lead to high treatment costs and even increase mortality. Effective preventive and therapeutic measures to reduce risks for implant-associated infections are thus in urgent need.

## 1. Introduction

With the rapid progress of modern medical technology, medical devices such as prosthetic joints, pacemakers, and catheters, etc. are playing an increasingly important role in medical care, which has completely changed medicine [1,2,3]. This is especially true in the field of orthopedics, because orthopedic implants are widely used for fracture fixation, deformity correction, joint replacement, and soft tissue anchorage [4].

However, due to the lack of self-cleaning ability, the use of implants also increases the risk of infection, which is the main reason for the failure of implants [5]. The infection caused by opportunistic pathogens can be cleared by the host’s immune system, while planting plants will expose the body to the risk of permanent colonization and planting failure. For example, when there is a foreign body at the surgical site, infected rabbits need much fewer *Staphylococcus aureus* cells than when there is no implant [6].

Biofilm can be defined as a structured microbial community of cells that are attached to a substratum and embedded in a matrix of extracellular polymeric substances (EPS) that they have produced [7]. Foreign implants placed in the patient’s body provide a colonization surface for bacteria so that bacteria and plasma proteins can adhere to it, and further form bacterial biofilm. Studies have indicated that bacterial cells in biofilm show different phenotypes from planktonic bacteria in terms of growth rate and gene transcription. Biofilms enable the bacteria embedded in them more resistant to antibiotics and less sensitive to host immune defense biological attack and are a source of persistent bacteremia as well as localized tissue destruction [8,9]. 

Staphylococci, with the leading species *Staphylococcus aureus* and Staphylococcus epidermidis, are the most common causes of implant-associated infections [10]. Whereas *S. aureus* has many mechanisms and virulence factors to evade the immune system, *S. epidermidis* must rely primarily on the ability to form a biofilm to survive in the host [11].

Clinical practice and experimental studies indicated clearly that in most cases, antibiotic therapy alone is insufficient to eradicate biofilm infection, and implants infected by bacteria usually require surgical resection [12,13]. In addition, it must be mentioned that each surgery to replace failed implants has a higher risk of recurrence (up to 30%) than that of the initial operation [14]. To solve these problems, great research progress has been made in the therapeutic approaches of biofilm, such as the studies on surface modification of implants, phage therapy, vaccine technology, and nanomedicines, but these new treatment methods need further preclinical and clinical validation before they are widely promoted for clinical use [15,16].

In this review, we focused on implant-associated infections in the orthopedic field, but some of the principles discussed also apply to other implants. We briefly discussed the microbial epidemiology of infected orthopedic implants, the formation process, and the function of biofilm. We also summarized the current strategies for the prevention and treatment of implant-associated infections, and emphasized the need to further explore biofilm physiology and carry out innovative research on anti-biofilm methods.

## 2. Microbial Epidemiology of Infections in Orthopedic Implants

Infection represents a terrible complication after orthopedic surgery [17]. There are significant differences in the etiology of implant-associated infection, which mainly depends on the type and anatomical location of the implant as well as the postoperative time [18]. Although there are some subtle differences in the time division of different types of orthopedic implant infection, the infection can be divided into three stages: early infection, late-acute infection, and late infection according to the time interval between surgery and clinical feature. Early infections are mainly caused by toxic microorganisms such as Staphylococcus aureus. Late-acute infections are usually caused by bacteria with low toxicity, such as coagulase-negative Staphylococcus. Late infections can be symptomatic infection evolved from an initially asymptomatic surgical infection or new infections caused by hematogenous seeding [19,20].

So far, there have been many epidemiological studies on orthopedic implant-associated infections. The results derived from the new study matched rather well with, and confirmed those of, the old epidemiological investigation [21,22,23,24]. In general, Gram-positive bacteria are the most common causes of orthopedic implant-associated infection, such as *Staphylococcus aureus* and Staphylococcus epidermidis. Aerobic Gram-negative bacilli, including Enterobacteria and P. aeruginosa, are less frequent causes of infection, and Anaerobic bacteria are seen far less frequently [19,24,25].

The largest study (thousands of cases containing 2524 adult patients with a diagnosis of prosthetic joint infection (PJI)) aimed at exploring the etiology of PJI was completed by Benito et al. in 2016. The results showed that the majority of cases of PJIs were caused by Aerobic Gram-positive cocci (77.7%), followed by aerobic gram-negative bacilli (27.6%). In addition, the rate of multidrug-resistant infections by resistant Gram-negative bacilli increased from 2003 through 2012, suggesting that reassessing empiric and specific antimicrobial therapy of PJIs could be required [26]. Recently Valérie Zeller et al. took 926 patients with PJI as samples, analyzed the microorganisms causing joint prosthesis infection, and compared their distribution according to the classification of PJI. Considering the 997 PJIs, staphylococci were the most frequently isolated microorganism (50%), followed by 16% streptococci, 11% Gram-negative rods, 8% anaerobic bacteria (predominantly Cutibacteriumacnes), and 9% polymicrobial infections. Early postoperative PJIs are characterized by a high rate of staphylococcal infections (57%), especially *S. aureus* (25%) and *S. epidermidis* (25%). For late-chronic PJIs, the most frequent microbes were low virulent microbes, such as coagulase-negative staphylococci (61%), with a high rate of *S. epidermidis* infections (35%). It is worth mentioning that among all PJI categories, hematogenous PJIs accounted for the largest proportion (35%) [21]. This observation is in contrast to spinal implant-associated infections where about 98% of them originated from perioperative colonization [22]. Besides, Murdoch et al. observed that internal fixation devices have a lower risk of hematogenous infection than joint prostheses [27]. This phenomenon may be caused by the continuous production of synovial fluid, which may expose the joint prosthesis to microorganisms that filtered from the blood to the joint space.

A study of 242 orthopedic patients showed that *S. aureus* was the main pathogen among infections associated with internal and external fixation systems, while *S. epidermidis* was the most prevalent bacterial species among infections associated with knee and hip arthro-prostheses [24]. In another study of 250 patients diagnosed with spinal implant-associated infections, Gram-negative bacteria were found mainly distributed in the lumbosacral segment because its anatomy is close to the genitourinary system and intestinal system [22].

## 3. Staphylococcal Biofilm Development

In orthopedic infection, the bacteria that cause implant failure are often not dispersed, but form biofilms [28], which occurs through a series of physical, chemical, and biological processes. Biofilm is a microbial community of bacteria that irreversibly attaches to a biomaterial surface and embedded in an abundant matrix of extracellular polymeric substances (EPSs) that they have produced [29].

The classic model of Staphylococcal biofilm development, which applies to *S. aureus* and S. epidermidis, is a complex process that can be divided into several main stages: (i) attachment and adherence, (ii) accumulation/maturation due to cellular aggregation and EPS production, and (iii) biofilm detachment (also called dispersal) of bacterial cells [19,30].

The solid–liquid interface between the implant surface and liquid media (such as water and blood) provides an ideal environment for the attachment and growth of microorganisms. Bacteria must first attach and adhere to biological or abiotic implant surfaces, which is a prerequisite for plant-related infection [29].

Although scientists have carried out many studies to study the exact mechanism involved in the process of bacterial adhesion, we still do not fully understand it. According to Gristina [31], when a foreign body in the form of biomaterial is implanted into a mammalian host, “a race for the surface” begins. The concept of “race for the surface” is proposed to describe the competition between host tissue cell integration and bacterial colonization at implant surfaces. If the host tissue cells occupy the available plant surface earlier, then tissue integration can be successfully carried out later, so the risk of infection will be reduced. However, if bacterial cells adhere to the surface of biomaterials before the host tissue cells, the surface is colonized by bacteria, which inevitably leads to implant-associated infection [32].

The attachment of bacteria to abiotic surfaces is instantaneous and reversible [4]. The physical forces involved in this process are hydrophobic, electrostatic, and Van der Waals forces, with bacteria behaving like colloidal particles. Some cell-surface multi-subunit protein polymers, known as pili or fimbriae, have an important role in the attachment to abiotic surfaces by many pathogenic bacteria [33]. Others specifically increase the direct adhesion of bacteria to implants by binding to host cell surface molecules and/or ECM components, such as collagen and fibronectin [34]. The autolysins AtlA [35,36] from *S. aureus* and the homologous protein AtlE [37] from *S. epidermidis* are bi-functional cell wall-anchored proteins, mediating binding to abiotic surfaces such as naked polystyrene. Some components on the surface of Staphylococcus, such as teichoic acid, also contribute to the adhesion to the surface of abiotic materials [38,39].

Actually, in vivo bacteria mainly encounter biotic surfaces composed of host matrixes including fibrinogen, fibronectin and collagen, because implants are rapidly covered by extracellular matrix (ECM) proteins when submerged in physiological fluids [34,40]. The process of plasma proteins attaching to the biomaterial’s surface is governed by the physical and chemical properties of the material surface such as polarity and roughness of the material, as well as the characteristics of the plasma proteins such as size and charge [41]. After being attached to the surface of biomaterials, these proteins form an ideal scaffold which provides a foothold for bacterial adhesion and subsequent proliferation [14]. For this purpose, staphylococci have evolved a series of cell wall-anchored (CWA) proteins, of which the best-characterized group is called MSCRAMMs (microbial surface components recognizing adhesive matrix molecules) [42,43], each specifically binding to ECM proteins covering the devices. Some examples of staphylococcal MSCRAMMs have been studied to full advantage, including fibronectin-binding proteins (FnBPA and FnBPB) [44,45,46,47], the fibrinogen-binding clumping factor A (ClfA) and clumping factor B (ClfB) [48,49,50,51], the serine–aspartate repeat protein family (Sdr) [52,53,54,55,56] and the collagen-binding protein (Cna) [57,58,59]. All MSCRAMMs contain a conserved C-terminal cell wall-targeting Leu-Pro-X-Thr-Gly (LPXTG) motif that covalently anchors the MSCRAMMs to the peptidoglycan by the specified protein, sortases (a membrane protein of Staphylococcus aureus) [60]. The mechanism is mainly that sortase catalyzes the cleavage of polypeptides between threonine and glycine in the LGXTG motif, and then mediates the formation of an amide bond between the carboxyl group of threonine and the amino group of peptidoglycan cross-bridges [61,62,63]. Some MSCRAMMs, such as *Staphylococcus epidermidis* surface protein serine-aspartate repeat protein G (SdrG), have a specific domain (SdrG_N2N3_) composed of N2 and N3 at the N-terminus, which binds to a ligand by the “dock, lock and latch” (DLL) mechanism [64,65]. *Staphylococcus aureus* surface protein can also bind to keratin [50,66], complement system protein [67,68], and other chains of fibrinogen [69] and collagen [57] through a similar DLL mechanism. For a more detailed overview of MSCRAMMs of Gram-positive cocci, readers can refer to a review by Foster [42].

After initial adhesion to a surface, these bacteria start to proliferate, gradually forming a multi-layered microcolony. At the same time, the bacteria also start to produce EPS, which eventually forms the biofilm matrix. The biofilm matrix is mainly composed of polysaccharide, protein, teichoic acid, and extracellular DNA (eDNA) as well as other molecules on the whole constituting a physical barrier between the microbial community and the extracellular environment [70,71].

*Staphylococcus epidermidis* and *Staphylococcus aureus* mainly produce polysaccharide intercellular adhesin (PIA) as the main polysaccharide of the biofilm matrix [10,72]. Due to the enzyme-catalyzed removal of partial N-acetyl groups, PIA has a cationic character [73], which allows electrostatic interaction with other negatively charged biofilm components (such as teichoic acid). The production of PIA is dependent on the intracellular adhesion (ica) operon, which contains four genes in sequence: icaA, icaD, icaB, and icaC [72,74,75]. Under the promotion of IcaD, the enzyme IcaA (an N-acetylglucosaminytransferase) synthesizes PIA oligomers with UDP-N-acetylglucosamine (UDP-GlcNAc) as the precursor. IcaC is involved in the synthesis of long-chain PIA and allows the translocation of PIA across the cell membrane [76]. IcaB is a secreted N-deacetylase that is responsible for the above-mentioned partial deacetylation of PIA [77]. In addition, icaR and tcaR are two negative regulatory genes that regulate the transcription of icaADBC operon [78]. Studies have suggested TcaR functions as a repressor only in the absence of IcaR [79,80].

Some studies have found that although several strains of *Staphylococcus epidermidis* and *Staphylococcus aureus* do not produce PIA, they can also form a biofilm with protein as the main component both in vivo and in vitro [81,82,83]. These staphylococcal surface proteins include fibronectin-binding proteins (FnBPA and FnBPB), serine aspartate repeat protein (SdrC), accumulation-associated protein (Aap), staphylococcal surface protein G (SasG) and biofilm-associated protein (Bap). Some MSCRAMMs not only play a role in bacterial adhesion, but also promote cell–cell interaction, resulting in increased biofilm formation. For example, the A domain of FnBPs and SdrC participates in homologous interactions and promotes the cell accumulation of staphylococcal cells during biofilm formation [53,82,84,85]. In the PIA-independent staphylococcal biofilm, there are also some specific bacterial surface proteins, such as accumulation-associated protein (Aap) from *S. epidermidis* and *Staphylococcus aureus* Aap homologue SasG (*Staphylococcus aureus* surface protein G). Specifically, mature Aap or SasG protein can mediate intercellular adhesion through several repeated B domains [86,87,88,89,90]. In addition, some studies have shown that PIA-dependent biofilm has fibrous structures that are absent in protein-dependent biofilm, and has higher strength and ductility than protein-based biofilm [91,92,93].

Extracellular DNA (eDNA) was more recently discovered to take part in supramolecular structures such as stable filamentous networks which work as a net to hold biofilm cells together [94]. Having an extremely important role, eDNA has been found to mediate horizontal gene transfer [95], limit the spread of antibiotics [96], promote antibiotic resistance phenotypes [97], interact with cells of the host immune system and regulate innate and cell-mediated immune responses [98], guide biofilm diffusion [99], and serve as a nutrient source during periods of nutrient deficiency [100].

With the proliferation of bacteria and the increase of EPSs, mature biofilm is finally formed, which is characterized by the presence of nonuniform growth of microcolonies surrounded by water channels that help to distribute nutrients and signal molecules [101]. Extensive in vitro studies have shown that the formation of water channels in mature biofilm is mainly completed by a polypeptide family called phenol-soluble modulins (PSMs) [102].

PSMs are a family of small peptides (~21–45 amino acids long) and have amphipathic, α-helical secondary structures. The smaller, ~20 amino acid peptides are grouped into the α-class and the longer ~45 amino acid peptides into the β-class of PSMs. PSMs exist in a monomeric state, where they have surfactant-like properties, and in a polymeric state, where they fold into an amyloid structure which can stabilize the biofilm structure [103].

The surfactant and lytic properties are believed to allow PSMs to disrupt non-covalent (electrostatic or hydrophobic) interactions between biofilm matrix components, thereby producing the characteristic fluid channels that are embedded in the biofilm matrix.

To date, eight different PSMs have been characterized in *S. aureus*, including four PSMα peptides, two PSMβ, δ-toxin, and PSM-mec. Compared with wide-type strain, *S. aureus* psm-mec mutants only show slightly decreased capacity to form biofilms [104], while the other types of isogenic *S. aureus* psm mutants produced more compact and extended biofilms which demonstrated less channel formation [105]. *S. epidermidis* produces six PSM peptides, PSMα, PSMβ1, PSMβ2, PSMδ, PSMε, and δ-toxin, but only the β-type PSMs were well investigated for their biofilm effects. Studies show that different concentrations of PSMβ play disparate roles in different stages of *S. epidermidis* biofilm cycle. At medium concentrations, PSMβ promoted the formation of channels and the growth of biofilm, while at high concentrations, they destroyed biofilm, thereby resulting in biofilm dispersal [91].

Finally, the third stage of biofilm development is the dispersal of bacterial cells from biofilm, which is an active process of bacteria escaping from the biofilm [106]. Transcriptome analyses of drip reactor-grown *P. aeruginosa* biofilms show that the steepening of the chemical concentration gradient of nutrient resources, oxygen, and waste seems to drive the dispersion of bacterial biofilm, during the development of biofilm [107]. In order to survive when there is a limitation of nutrients or to establish infection at distant sites in the human body, some bacterial cells detach from the biofilm individually or in agglomerates, leaving behind eroded biofilms with central voids.

Compared with their biofilm and planktonic counterparts, dispersed bacteria express high levels of genes encoding virulence factors and genes important for motility and adhesion, which may lead to subsequent more serious infection [108].

Mechanical stress or shear force caused by blood flow and equipment flushing is considered to be the main mechanism limiting the overall biomass accumulation of the biofilm [109]. In addition, various enzymes and PSMs produced by bacteria that degrade the components of biofilm matrix all contribute to the stage of biofilm dispersal.

The important role of PSM in biofilm dispersal has been confirmed in vivo experiments. In a murine model of device-related infection caused by Staphylococcus epidermidis, bacteria harvested from body fluids and lymph nodes were overwhelmingly more of the WT than psmβ mutant strain, indicating PSMβ contributes to the dissemination of biofilm-associated infection [91].

With a deeper understanding of the matrix components of biofilm, the enzymes that degrade EPSs have also been widely studied. A variety of enzymes, including proteases, glycoside hydrolases, and nucleases, have been tested for their ability to degrade biofilm and promote biofilm dispersal [110]. The relative importance of each enzyme is decided by the strain-specific components of the biofilm matrix.

*S. aureus* produces and secretes four extracellular proteases of different protease families that may digest proteinaceous matrix components: metalloprotease aureolysin (Aur), serine protease SspA (also named V8 protease), and two cysteine proteases SspB and ScpA. Some of these proteases have been identified to degrade specific matrix proteins. For example, SspA digests FnBPs and Bap, whereas Aur degrades Bap as well as ClfB [110,111,112].

The biofilm matrix component eDNA is degraded by nuclease. To our knowledge, *S. aureus* nucleases Nuc1 and Nuc2 modulate biofilm formation [113,114].

A large number of studies have been carried out on the decomposing of extracellular polysaccharides by glycoside hydrolases as a means of dispersing biofilms. So far, an enzyme secreted by staphylococcus that can degrade PIA has never been found. Dispersin B, a glycoside hydrolase produced by Actinobacillus actinomycetemcomitans, has been shown to degrade PIA by hydrolyzing β-1,6-glycosidic linkages [115]. This enzyme is effective against biofilms made by a variety of bacteria, including *S. aureus* and *S. epidermidis* [116]. Cellulase is a glycoside hydrolase produced by multiple microbes that hydrolyzes the β-1,4-glycosidic linkage. It has been shown to cause the dispersal of *S. aureus* biofilms [117].

Dispersion is also associated with increased bacterial cell death in local hypoxic areas within the biofilm. Nitric oxide (NO) produced by bacteria through anaerobic denitrification react with superoxide to generate the cell-toxic radical ONOO^−^. ONOO^−^ leads to cellular damage and cell lysis, which has a synergistic effect with enzyme-mediated destruction of biofilm matrix [118].

## 4. The Role of Staphylococcal Biofilm during Infection

The formation of biofilms creates significant challenges for treating implant-associated infections because bacteria in biofilms display unique characteristics and behavior compared with their planktonic counterparts, with the hallmark characteristics including an innate resistance to antimicrobials and host immune defenses.

## 5. Antibiotic Tolerance

It has been reported that the tolerance of bacteria in biofilm to antibiotics is 10- to 1000-fold higher for that of their planktonic counterparts [119]. Notably, bacteria with increased resistance in biofilm have the same minimum inhibitory concentration as the planktonic controls after dispersing from the biofilm. This indicates that the existence of biofilm plays a significant role in the enhancement of bacterial antibiotic tolerance [120]. The increase of antibiotic resistance of bacteria in biofilm is the comprehensive result of different mechanisms, including slow growth and reduced metabolic rates [121], the existence of persister cells [122], and the barrier effect of the biofilm matrix [123].

Compared with planktonic bacteria, bacteria growing at different locations in the biofilm community experience different concentration gradients of oxygen, nutrient resources, and metabolic wastes, as well as extracellular signal molecules [124], which will lead to changes in the metabolic rate of bacteria within the biofilm. In addition, the higher bacterial cell density in the biofilm community contributes to the transfer of tolerance genes between bacterial cells, which promotes the formation of subpopulations with different specialties in the community [125]. It is considered that an important reason why antibiotics cannot effectively remove biofilm bacteria is that biofilm communities can harbor tolerant and persister cells [126]. They contribute to the formation of chronic infections and complicate the treatment of implant infections. Common targets of antibiotics, such as RNA polymerase and cell-wall biosynthetic enzymes, represent low activity in metabolically inactive cells, and thus, slow-growing cells can survive transient or periodic antibiotic treatment and regrow when antibiotics are withdrawn. Unlike antibiotic resistance, antibiotic resistance can be caused by many, not a few, gene mutations, so tolerance mutations occur more frequently than resistance mutations. Persister cells are subpopulations of phenotypically dormant cells, originated from part of pre-existing non-growing cells or cells that are dormant at rest during stationary phase. Mutations in genes encoding tRNA synthetase, ribose-phosphate diphosphatekinase and toxin antitoxin (TA) systems are all tightly associated with induced cell dormancy, persister cell formation, and antibiotic tolerance [127].

Another factor promoting to development of antibiotic tolerance is the biofilm environment. Biofilm matrix containing numerous charged molecules (e.g., proteins, glycoproteins, polysaccharide, eDNA, etc.) can bind charged antimicrobial agents and provide a physical barrier that protects the microorganisms from immune components [128]. The specific interactions between the components of the biofilm matrix give rise to the necessarily global biofilm mechanical properties, which can protect the bacteria from external forces such as fluid shear and ensure the biofilm community remains attached to a surface. Moreover, the strength of the in vivo biofilm scaffold exceeds the maximum mechanical stress that neutrophils can exert, which makes it difficult for bacteria in the biofilm to be completely cleared by the immune system. Therefore, the biofilm matrix scaffold can still harbor tolerant or persister cells that can regrow and cause chronic infection even if the majority of actively growing pathogens in biofilm have been killed by antibiotics.

## 6. Immune Evasion

Bacterial invasion commonly stimulates a strong inflammatory response, which includes involves complement activation, recruitment of phagocytes (most importantly neutrophils), and subsequent killing of the pathogen. Neutrophils recruited to the infected site can kill bacteria by phagocytosis, degranulation of antimicrobial substances into the environment, or neutrophil extracellular trap (NET) formation [129]. In the past, it has been proposed that the resistance of bacteria in biofilm to immune cells is mainly due to the physical barrier effect of biofilm matrix [130]. However, in vitro studies have suggested that human leukocytes are able to penetrate biofilm matrix effectively and bacteria in biofilms are not inherently protected from phagocytic cells [131].

*S. aureus* relies on several immune-evasion proteins and cytolytic toxins that kill immune cells directly to evade the host immune response, and by skewing host immunity toward an anti-inflammatory, pro-fibrotic response instead of a pro-inflammatory, bactericidal response that favors staphylococcal persistence [132,133]. Fibronectin (Fn) binds to fibronectin-binding proteins (FnBPs) on the surface of *Staphylococcus aureus* and to α5β1 integrin on the surface of osteoblasts, thus forming a FnBP–Fn–α5β1 bridge that triggers the invasion of osteoblasts [134]. Inside the host cells, *S. aureus* is not vulnerable to extracellular antibiotics and immune cells. In addition, *S. aureus* secretes multiple proteins to prevent antibody opsonization and interfere with the complement system. For example, Staphylococcal protein A and Staphylococcal binder of immunoglobulin (Sbi) block Fc receptor-mediated phagocytosis by binding IgG in the wrong orientation [135]. Extracellular adherence protein (Eap) blocks the formation of lectin and classical pathway C3 convertases, thereby blocking C3b deposition and C5a production [136]. *S. aureus* also secretes toxins that induce immune cell lysis by disrupting the cell membrane [137]. Some toxins specifically target leukocytes via the interaction with receptors, including alpha-hemolysin (Hla) and bicomponent leukocidins Panton-Valentine leukocidin (PVL), gamma-hemolysin (HlgAB, HlgCB), leukocidinED (LukED), and leukocidinAB/GH (LukAB/GH).

Compared with *S. aureus*, *S. epidermidis* has fewer mechanisms to evade immune defenses, and its pathogenicity relies mainly on biofilm formation [138]. PIA by deacetylation of its poly-N-acetylglucosamine molecule forms a sort of positively charged “capsule” around S. epidermidis, which has been shown to protect against neutrophil phagocytosis and AMPs presumably by charge repulsion and physical barrier function [77,139]. Animal experiments and the observation of human *Staphylococcus epidermidis* biofilm infection showed that the immune response of wild-type *Staphylococcus epidermidis* strains to biofilm infection was generally weakened compared with the immune response from ica-negative isogenic mutants [140,141]. It is the low immunogenicity of *S. epidermidis* that makes it only diagnosed in a late stage of infection, when there is a mature biofilm.

## 7. Prevention and Therapy

Over the last decade, with the steady increase of the impact of multidrug-resistant bacteria in healthcare-associated infections, plant biofilm-associated infections are becoming a public health problem of growing importance worldwide. In the Unites States, the annual morbidity and mortality of biofilm-associated infection are very high, with over $18 billion in direct treatment costs spent on these infections [142]. Clinical management of biofilm-associated infections is a challenging and multifactorial problem. Although various strategies have been adopted, so far there is no method that can completely avoid biofilm-related infection. The risk of surgical site infections in orthopedic surgeries can be effectively reduced with preoperative interventions for patients [143].

A simple example of preoperative interventions is optimizing preoperative medical conditions. Systemic prophylaxis in a 24 h regimen, especially with high-risk patients, is also one of the strategies to prevent infection.

Anti-infective biomaterials currently represent a main preventive strategy [144,145]. The causes of implant-associated infection are multifactorial, and bacterial adhesion to the implant surface is the premise of infection. The ideal implant surface would be one that minimizes bacterial adhesion, confers effective sterilization, and has no interference with bone healing and osteointegration [20]. Great efforts have been made to deal with the adhesion of bacteria to implants by modifying its surface to lower adhesive features or by the design of antibacterial coatings [4,146].

Implant coatings can be divided into passive and active according to the mode of action. Passive coatings reduce bacterial adhesion by altering the implant surface chemistry and/or surface structure modification without local release of fungicides to surrounding tissues. The active coating releases pharmacologically active pre-incorporated bactericidal agents, such as antibiotics, preservatives, metal ions, non-metal ingredients (e.g., iodine, selenium) and functional peptides, thereby reducing infection [15]. Unfortunately, these approaches do not result in complete clinical success, possibly due to the fact that implants are prone to be covered by plasma proteins quickly so that the anti-biofilm compounds are masked and their efficacy diminished.

Although the purpose of the abovementioned strategies is to prevent the formation of biofilm, it is still necessary to deal with the biofilm that has already been formed. Currently, the only remedy to ensure complete resolution of the infection is surgical excision of the implant, thorough debridement of the peri-implant environment, and prolonged antimicrobial therapy [147], which not only causes extreme suffering for patients but also a huge economic burden on the healthcare system. Novel strategies have been researched to eradicate the biofilm in situ, but so far, no method of in-situ biofilm eradication that would provide a working alternative to the use of antibiotics has yet been taken to clinical use. 

Rifampicin, an inhibitor of bacterial RNA polymerase, is an important antibiotic drug for the treatment of staphylococcal prosthetic joint infections, as it is able to penetrate staphylococcal biofilms [148]. Owing to the risk of the quick development of rifampicin-resistant isolates, rifampicin should be used with other antibiotics [149]. In addition, fluoroquinolones show the characteristics required to effectively eliminate gram-negative bacilli biofilm [150].

In the following, some novel strategies to eradicate biofilm in situ are presented, but so far, there has been limited translation into clinical practice. 

Bacteriophages (phages), viruses that specifically infect and kill their bacterial hosts, have been investigated as an alternative to antibiotic treatment due to the growing degree of anti-microbial resistance, and they have been studied in some clinical trials demonstrating safety and possible efficacy [151]. The bactericidal effect of phage depends on phage-derived lysine, which can decompose peptidoglycan, the main component of the bacterial cell wall, thereby inducing cell lysis.

A recent study in a murine model of PJI suggested that the combination of phages with antibiotics may increase the removal of bacteria. Both the orthopedic wires coated with phage K or coated with linezolid alone demonstrate a modest decrease in the bacterial load, but the use of dual coated implants incorporating lytic phage as well as linezolid presents a stronger antibacterial effect [152]. Therefore, according to the different mechanisms of killing bacteria between antibiotics and phages, researchers speculated that the combination of phages and antibiotics may be more effective in controlling bacteria than one of them alone [153]. Moreover, Ferry et al. successfully treated multiple patients with recurrent *S. aureus* and *P. aeruginosa* PJI by combining antibiotic therapy with phage therapy, and preserved their prosthetic joints. This indicates that the combination of phage therapy and antibiotic therapy may become a salvage measure for preserving prosthetic joints of patients with recurrent PJI [154,155]. However, it seems that this approach is only experimental because antibiotics still have to be maintained during the phage therapy. 

Another novel treatment strategy is the use of the vaccine, but all clinical trials of anti-staphylococcal vaccines have failed [156]. Nevertheless, several vaccine candidates have been investigated that specifically target which biofilm matrix components are present in the biofilm growth. For example, immunization with PIA reduced the consequences of osteomyelitis infection from PIA-producing intercellular adhesion in an *S. aureus* periprosthetic osteomyelitis rat model [157]. As for S. epidermidis, there appears to have been some preclinical success with anti-Ses antibodies or active vaccination using recombinant truncated Ses in subcutaneous catheter mouse models [158].

Materials of nano size with unique physical and chemical properties, strong bactericidal activity, and specific mechanisms have shown great potential for eradicating resident bacteria and pathogenic biofilm [159]. According to the elemental composition, these nanomaterials can be divided into organic (e.g., metal and metallic oxide nanoparticles) and inorganic (e.g., lipid nanoparticles and smart nanomaterials) platforms. In addition to their multiple mechanisms, such as physical damage, oxidative stress, thermal damage, and so on, nanomaterials’ high surface-to-volume ratios and multivalent interactions enable them act as carriers for antibiotics or drug combination loading that may produce synergistic anti-biofilm efficacy. Critically, nanomaterials can be designed to become activated in response to unique biofilm pathologic microenvironments (e.g., pH, light, temperature, hypoxia, and enzymatic activation) so as to accurately carry, retain, and release drugs when and where needed most. Moreover, these dispersed nanoparticles can be assembled on platforms suitable for therapeutic purposes as well as in the form of various devices (bandages, catheters, implant coatings, etc.) to prevent and control infections [160].

Although nanoparticles have shown good inhibitory effects on biofilm formation, studies on the inhibitory effect of nanoparticles on persister bacteria in biofilm are still rare. Different from traditional antibacterial drugs, smart nanoparticles with new anti-biofilm mechanisms (such as photothermal therapy (PTT) and photodynamic therapy (PDT)) might be a more promising method to kill the persister cells [161]. PTT relying on the conversion of near-infrared light (NIR) into local heat by photothermal agents destroys the integrity of bacteria or the structure of biofilm through local hyperthermia, and can promote the diffusion of photosensitizers to biofilm at the same time. The main disadvantage of this strategy is that bacteria can only be effectively removed at higher temperatures. However, when high temperature kills the formed biofilm, it will have a huge negative impact on the surrounding healthy tissues. Although healthy tissues can withstand temperatures above 50 °C for a long time, the antibacterial effect is limited at such temperatures. If the sensitivity of the biofilm to heat is improved by using a safe photosensitive heat (below 50 °C), the biofilm formed on the surface of the implant can be eradicated without damaging the surrounding tissue [162]. PDT is another promising way. Nano materials or photosensitizers stimulate reactive oxygen species (ROS) under near-infrared or visible light irradiation, which will destroy the integrity of bacterial cell membrane, and lead to the enhancement of bacterial sensitivity to heat and bacterial death. The ROS produced by nano materials or photosensitizers under near-infrared or visible light irradiation will destroy the integrity of bacterial cell membrane, and improve the thermal sensitivity of the biofilm [163]. Therefore, the combination of photothermal therapy and photodynamic therapy in the treatment of implant related infections is a hot research field today. Recently, a study used a red-phosphorus-IR780-arginine-glycine-aspartic-acid-cysteine coating on titanium bone implant with irradiation of NIR light (808 nm) to eradicate biofilms by combining PTT and PDT. Red phosphorus can be used as an efficient photothermal coating for its great biocompatibility. The temperature sensitivity of *S. aureus* biofilm is enhanced in the presence of ROS produced by IR780. At the same time, arginine-glycine-aspartic-acid-cysteine decorated on the surface of the implant can facilitate the integration of implant materials and bone tissue. Finally, the technique demonstrated a rapid bactericidal effect that was biosafe at a safe temperature (~50 °C) [164].

Driven by the motivation to write high impact articles, researchers have created a large number of anti-biofilm therapies based on nanotechnology, and have carried out in vitro experiments or animal experiments for verification [165]. Unfortunately, there are still many challenges that need to be solved before nanomaterials are applied to clinical practice. Off-target toxicity of nanomaterials should be paid attention to first [166]. Metal and inorganic nanomaterials can be absorbed within the gastrointestinal tract. Although the bioavailability is very low and nanomaterials may degrade metabolism, the chronic off-target effect caused by them may be long-lasting. For example, magnetite iron oxide nanoparticles increase endothelial permeability by producing robust radicals, which may lead to hepatotoxicity and nephrotoxicity. Therefore, it is necessary to use nanoparticle systems prepared from degradable biomaterials as much as possible. Next, due to the physiological differences between animals and humans, it is still open to question whether the nanomedicines verified as safe by animal experiments can be used in humans. In addition, the production cost of nanoparticles is high, so the economic benefits brought by using nanotechnology to solve clinical problems cannot be accurately predicted at present. Based on the abovementioned challenges, we believe that the future direction should be to effectively eradicate biofilms, minimize toxicity and drug resistance, and promote low-cost and high-efficiency clinical applications.

## 8. Conclusions

Despite the rapid development of modern medical treatment, implant related infection is still a serious clinical problem faced by surgeons. As mentioned above, the implants implanted in the body compromise host defenses and provide a site for bacteria to adhere to and colonize it. Staphylococcus is the most common bacterium causing implant related infection, and the main pathogenic microorganisms are *Staphylococcus aureus* and Staphylococcus epidermidis. These bacteria can adhere to the surface of implants and form biofilm through various mechanisms. Bacteria growing in biofilm have stronger antibiotic resistance than planktonic bacteria, which leads to the fact that antibiotic treatment alone is not enough to eradicate biofilm. Therefore, different treatment strategies need to be combined and integrated to fight implant-associated infections.

Currently, the only remedy to ensure complete resolution of the infection is surgical excision of the implant, thorough debridement of the peri-implant environment, and prolonged antimicrobial therapy, which causes heavy physical, emotional, and financial burdens.

Novel strategies have been researched to prevent and eradicate the biofilm in situ, but so far, no method of in-situ biofilm eradication that would provide a working alternative to the use of antibiotics has yet been taken to clinical use.

Given that biofilm-related infection of implants is a major public health problem that has not yet been solved, there is an urgent need to carry out more basic and clinical research on Staphylococcus and other related pathogens infecting implants. Importantly, the in vivo evaluation of the therapeutic effect of anti-biofilm deserves more attention.

## Data Availability

Not applicable.
